# Postglacial colonization history reflects in the genetic structure of natural populations of *Festuca rubra* in Europe

**DOI:** 10.1002/ece3.4997

**Published:** 2019-03-04

**Authors:** Maria von Cräutlein, Päivi H. Leinonen, Helena Korpelainen, Marjo Helander, Henry Väre, Kari Saikkonen

**Affiliations:** ^1^ Natural Resources Institute Finland (Luke) Helsinki Finland; ^2^ Biodiversity Unit of University of Turku Turku Finland; ^3^ Department of Agricultural Sciences, Viikki Plant Science Centre University of Helsinki Helsinki Finland; ^4^ Department of Biology University of Turku Turku Finland; ^5^ Botanical Museum, Finnish Museum of Natural History University of Helsinki Helsinki Finland; ^6^ Present address: Department of Agricultural Sciences, Viikki Plant Science Centre University of Helsinki Helsinki Finland; ^7^ Present address: Biodiversity Unit of University of Turku Turku Finland

**Keywords:** CpDNA, *Epichloë festucae*, *Festuca rubra*, genetic structure, postglacial colonization history, symbiotic microbes

## Abstract

We conducted a large‐scale population genetic survey of genetic diversity of the host grass *Festuca rubra* s.l., which fitness can be highly dependent on its symbiotic fungus *Epichloë festucae*, to evaluate genetic variation and population structure across the European range. The 27 studied populations have previously been found to differ in frequencies of occurrence of the symbiotic fungus *E. festucae *and ploidy levels. As predicted, we found decreased genetic diversity in previously glaciated areas in comparison with nonglaciated regions and discovered three major maternal genetic groups: southern, northeastern, and northwestern Europe. Interestingly, host populations from Greenland were genetically similar to those from the Faroe Islands and Iceland, suggesting gene flow also between those areas. The level of variation among populations within regions is evidently highly dependent on the postglacial colonization history, in particular on the number of independent long‐distance seed colonization events. Yet, also anthropogenic effects may have affected the population structure in *F. rubra*. We did not observe higher fungal infection rates in grass populations with lower levels of genetic variability. In fact, the fungal infection rates of *E. festucae* in relation to genetic variability of the host populations varied widely among geographical areas, which indicate differences in population histories due to colonization events and possible costs of systemic fungi in harsh environmental conditions. We found that the plants of different ploidy levels are genetically closely related within geographic areas indicating independent formation of polyploids in different maternal lineages.

## 1 INTRODUCTION

The genetic structure of several plant taxa occurring in Europe shows evidence of postglacial range expansion and colonization from refugia located in the peninsulas of Southern Europe, including Iberia, Italy, and the Balkans, and possibly also Caucasus and the Caspian sea (Hewitt, 1999). Populations located near glacial refugia are typically characterized by high amounts of genetic variation, accumulation of private alleles during the glacial advance (Hewitt, 1999; Taberlet, Fumagalli, Wust‐Saucy, & Cosson, 1998), and high degree of genetic distinctiveness (Jimenez‐Mejías, Luceno, Lye, Brochmann, & Gussarova, 2012; Schönswetter & Tribsch, 2005; Tribsch, Schönswetter, & Stuessy, 2002). Moreover, genetically distinct, small glacial refugia, nunataks, can increase genetic diversity, as they may contain unique alleles in spite of likely drastic reductions in genetic diversity as a result of genetic drift (Brochmann, Gabrielsen, Nordal, Landvik, & Elven, 2003 and references therein; Westergaard, Alsos, Popp et al., 2011; Parducci et al., 2012; Eidesen et al., 2013).

Rapid range expansion from the refugia has often been found to be associated with a founder effect and, consequently, decreased genetic diversity and genetic drift (Eidesen et al., 2013; Hewitt, 1999; Petit et al., 2003). Additionally, human‐mediated dispersal has potentially affected the genetic structure of several grass species used in the agriculture in Europe, as observed in European forage grass species (Balfourier, Imbert, & Charmet, 2000). As a result of admixture, increased genetic diversity has been detected in contact zones, where different natural or human‐induced colonization routes meet (Petit et al., 2003; Taberlet et al., 1998). As a result of multiple introductions from different origins and recombination between divergent genomes, species can form admixed, possibly highly variable populations.

The family Poaceae, commonly referred to as grasses, is the most successful and economically important plant family, with global occurrences ranging from extremely dry and cold arctic to moist and hot tropical environments (Linder, Lehmann, Archibald, Osborne, & Richardson, 2018). The success of grasses is proposed to be caused by their superior colonization ability (Inda, Segarra‐Moragues, Müller, Peterson, & Catalán, 2008), due to efficient dispersal and establishment abilities, rapid population growth, tolerance of disturbances, and phenotypic plasticity (Linder et al., 2018). In addition, symbiotic microbes and polyploidization events have been shown to be important for the worldwide success of grasses (Te Beest et al., 2012; Clay & Schardl, 2002; Linder & Barker, 2014; Saikkonen, Wäli, Helander, & Faeth, 2004). Microbial endosymbionts are very common and fundamental in many grass species and can affect stress tolerance of the host species (reviewed by Cheplick & Faeth, 2009; Rodriquez, White, Arnold, & Redman, 2009; Malinowski & Belesky, 2000). The pooid grasses often harbor systemic filamentous fungi *Epichloë* (Clavicipitaceae). Many studies have demonstrated that a grass host benefits from *Epichloë* due to, for example, increased resistance to drought, herbivores, and pathogens (Bazely et al., 1997; Clay & Schardl, 2002; Wäli, Ahlholm, Helander, & Saikkonen, 2007; Wäli, Helander, Saloniemi, Ahlholm, & Saikkonen, 2009). Symbiotic fungi of grass species can contribute to the sustainable agricultural system by reducing the need and use of synthetic pesticides (Gundel, Perez, Helander, & Saikkonen, 2013; Kauppinen, Saikkonen, Helander, Pirttilä, & Wäli, 2016; Saikkonen, Faeth, Helander, & Sullivan, 1998). *Epichloë* can also increase a plant's allocation to female reproductive organs (Saikkonen, Wäli, & Helander, 2010), but it can also decrease host fitness in some environmental conditions (Ahlholm, Helander, Lehtimäki, Wäli, & Saikkonen, 2002) and prohibit the sexual reproduction of the host via stromata production around the developing inflorescences (Clay, 1988; Clay & Schardl, 2002; Saikkonen et al., 1998, 2004; Schardl, 2001). However, the associated horizontal transmission by sexual spores seems to be less frequent than vertical transmission via host seeds (Dirihan et al., 2016; Wäli et al., 2007; Zabalgogeazcoa, Vázquez de Aldana, García Criado, & García Ciudad, 1999).

The symbiosis is facultative for the host and fitness effects can vary from antagonistic to mutualistic depending on the prevailing environmental conditions while *Epichloë* is completely dependent on its host in terms of survival, nutrition, reproduction, and transmission abilities (Clay & Schardl, 2002; Saikkonen et al., 1998). Genetic variability of both the host and fungus is a prerequisite for populations to adapt to changing selection pressures (Gundel et al., 2010). Vertical transmission of *Epichloë* can form highly specialized maternal lineages, since only one fungal genotype is typically transmitted to the seed progeny. In seeds, cross‐fertilization of the host introduces new genetic combinations of host genotypes to *Epichloë,* and can cause a genetic mismatch between the systemic fungus and the host, if the fertilizing pollen is genetically distant, resulting in incomplete transmission (Gundel et al., 2010; Saikkonen et al., 2010, 2004).

In this study, our main two objectives are to gain further knowledge of genetic diversity patterns and seed flow in *F. rubra* s.l. occurring in southern and northern Europe, and of the genetic diversity patterns of host populations with differing infection rates of symbiotic fungi. We hypothesized that genetic diversity would be reduced, especially at the edges of the European range due to potential founder effects, bottlenecks, and selection, while greater diversity would be expected near refugial areas and contact zones. We expect to observe higher fungal infection rates in grass populations with lower levels of genetic variability, and vice versa, because of possible genetic mismatches between the host and the fungus. We aimed at answering the following questions: (a) How is genetic diversity distributed among populations and regions? What is the level of geographic population genetic structuring and how has the postglacial colonization shaped the geographic distribution of haplotypes? (b) Is there association between *E. festucae* infection rates and the levels of genetic diversity in the host plant populations? (c) Does genetic structure within ploidy levels differ among geographic areas?

## 2 METHODS

### 2.1 Study system

We used perennial, rhizomatous grass *Festuca rubra *L., s.l. (red fescue, Poaceae) as a model for estimating genetic variability and population structure across the Europe range to evaluate the genetic resources to create novel plant gene combinations for use of crop varieties. *F. rubra* is an important turf grass that is widely cultivated in temperate regions (Gould & Shaw, 1983; Hand, Spangenberg, Forster, & Cogan, 2013; Inda et al., 2008). The western Mediterranean *F. rubra* group is estimated to have diverged during Pleistocene (c. 1.6 MYA; Inda et al., 2008). Inda et al. (2008) suggest in their biogeographical study on Loliinae that *F. rubra* has experienced postglacial expansion into northern Eurasian latitudes and more recently colonized holarctic areas from Eurasia. Wind pollinated, outcrossing, and self‐incompatible *F. rubra* reproduces sexually by seeds and effectively by asexual vegetative (tillers) and pseudoviviparous propagules (Ahlholm et al., 2002; Dirihan et al., 2016). Clonal tillering is the predominant reproduction strategy facilitating the efficient establishment of a genotype within a habitat, but only a small part of the tillers produce inflorescences yearly (Jónsdóttir, 1991; Saikkonen et al., 2010). There is no marked seedbank of *F. rubra *(Wäli et al., 2009), and thus, environmental conditions at the time of seed ripening are likely to determine germination rates and the survival of seedlings. The success of *F. rubra* can be connected with the systemic and vertically transmitted symbiotic fungus, *Epichloë festucae* Leuchtm., Schardl & Siegel (Clavicipitaceae; Ahlholm et al., 2002; Saikkonen et al., 2010; Wäli et al., 2007; Wäli et al., 2009). *E. festucae* infections have been observed to be common in *F. rubra* in dehesa grasslands in Spain (Dirihan et al., 2016; Zabalgogeazcoa et al., 1999) and in northern latitudes in subarctic regions (Dirihan et al., 2016; Wäli et al., 2007). Sexual reproduction of *E. festucae* (stroma formation, choke disease) has regularly been observed in Spain (Zabalgogeazcoa et al., 1999; pers. comm. Pedro Gundel), but not in the subarctic (Wäli et al., 2007).

### 2.2 Plant material

**Figure 1:**
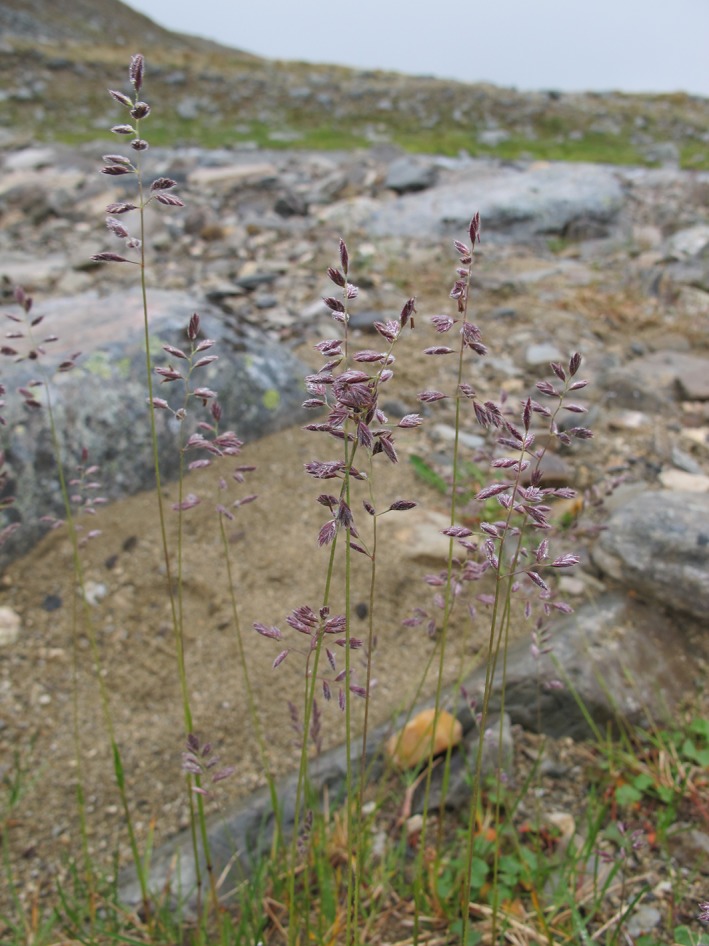
*Festuca rubra*, red fescue, occurs abundantly in the mountains of northern Finland

**Figure 2:**
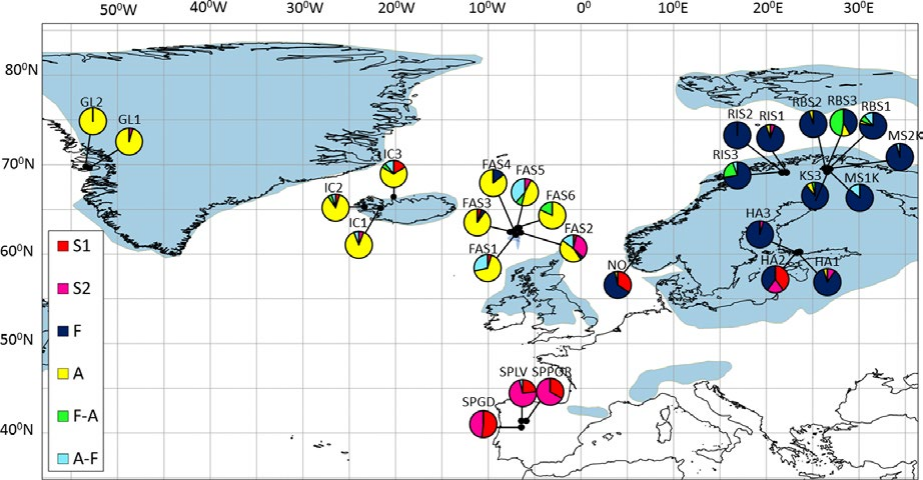
The locations of sampled populations and the distribution of the genetic clusters within sampled natural populations of *Festuca rubra*. Blue regions indicate permanent ice cover during the last glacial period (25,000–15,000 years ago; Ray & Adams, 2001). BAPS clusters: S1 = *Spain1‐group, *S2 = *Spain2‐group, F* = *Fennoscandia‐group*, A = *Atlantic‐group*, *F–A‐cluster *and *A–F‐cluster*. Population locations: Spain: SPGD, SPLV, and SPPOR; Norway: NO; South Finland, Hanko: HA1, HA2, and HA3; North Finland, Kilpisjärvi: RIS1, RIS2, and RIS3; North Finland, Kevo: MS1K, MS2K, KS3, RBS1, RBS2, and RBS3; the Faroe Islands: FAS1, FAS2, FAS3, FAS4, FAS5, and FAS6; Iceland: IC1, IC2, and IC3; Greenland: GL1 and GL2. Population and sample information can be found in Supporting information Appendix S1

A total of 603 *F. rubra *s.l. plants were collected from 27 populations over a wide range of environments and latitudes from the following geographic sites across Europe: Spain, the Faroe Islands, Iceland, Greenland, southern Norway, and three locations in Finland, including Hanko from southern Finland and Kilpisjärvi and Kevo from northern Finland (Figure 1; Figure 2; Supporting information Appendix S1). The samples included four closely related taxa identified by local taxonomists: *F. rubra* L. subsp. *rubra,* *F. rubra* subsp. *arctica* (Hack.) Govor. (synonym *F. richardsonii* Hook.) and their hybrid (*rubra* x *arctica*) in northern Europe, and *F. rothmaleri* (Litard.) Markgr.‐Dann. (synonym *F. rubra* subsp. *rothmaleri* Litard.) in Spain (Foggi & Müller, 2009; Inda et al., 2008; Lu, Chen, & Aiken, 2006). The taxonomic identities of the plant samples in the regions are given in Supporting information Appendix S1. The nuclear ITS region (ITS1‐5.8S‐ITS2) is commonly used for phylogenetic analyses in Loliinae (Inda et al., 2008). The ITS sequence data of the same samples used in this study revealed no sequence differences among the *F. rubra *subspecies and their hybrids occurring in Finland and northern Atlantic islands, while minor ITS sequence differences were found between *F. rubra *taxa and *F. rothmaleri* (Saikkonen et al., *in prep*). Hereafter, we refer to the studied taxa as *F. rubra*.

The sampling procedure and *E. festucae* detection (infected E+/uninfected E−) has been described in Dirihan et al. (2016). The frequency of endophyte infections (E+) was 44.9% in our sample set (*N* = 566). The percentages of the endophyte infections varied from 0% to 82.5% among regions and from 0% to 95.8% among populations (Supporting information Appendix S1; see also Dirihan et al., 2016 for their larger sample set). The frequency of endophyte infections was 82.5%, 73.1%, 55.4%, and 38.2% in Spain, Kevo in northern Fennoscandia, Iceland, and the Faroe Islands, respectively. No infections were observed in southern (Hanko) and northern (Kilpisjärvi) Fennoscandia, and only one plant was infected in Greenland and Norway each. In addition to variation in *E. festucae* infections, the studied populations were found to include plants with different ploidy levels, and the proportions of tetraploids (2n* *= 4x = 28), hexaploids (2n = 6x = 42), and octoploids (2n = 8x = 56) were 12.7, 82.2, and 5.1%, respectively, in our data set (*N* = 566; Supporting information Appendix S1; see also Dirihan et al., 2016 for their larger sample set).

Genomic DNA of *F. rubra* was extracted from fresh leaves of *F. rubra* using the E.Z.N.A. Plant DNA Kit (Omega Bio‐Tek, Norcross, Georgia, USA). A total of thirteen highly polymorphic chloroplast microsatellite markers (cpSSRs) developed for *F. rubra* by von Cräutlein, Korpelainen, Helander, Väre, and Saikkonen (2014) were used for the genetic characterization of the whole sample set (*N* = 603). Chloroplast DNA markers were chosen for the study because they are haploid and maternally inherited in angiosperms, which allows us a direct interpretation of seed‐mediated gene flow (for review, see Provan, Powell, & Hallingsworth, 2001; Ebert & Peakall, 2009) and a comparison of dispersal patterns of *F. rubra* and its symbiotic fungus*,* which are transmitted vertically along with seeds of *F. rubra*. Drawbacks of cpSSR markers include primarily size homology; however, it is not a significant problem at the intraspecific level (Ebert & Peakall, 2009; Estoup, Jarne, & Cornuet, 2002; Navascués & Emerson, 2005; Provan et al., 2001). The forward primers of each cpSSR primer pair were end‐labeled with two different phosphoramidite fluorescent dyes, either 6‐FAM or HEX. The samples were analyzed by multiplexing markers with different labels and expected fragment sizes (2–3 samples per PCR reaction). All primer pairs produced bands that matched the expected sizes. PCR amplifications were performed, as described in von Cräutlein et al. (2014). Each genotyping plate included individuals from several populations, and negative and positive controls. In the case of rare alleles, PCR amplifications were repeated to ensure the existence of rare alleles. The PCR products were run on an ABI 3130xl DNA Sequencer using the GeneScan 500 ROX Size Standard (Applied Biosystems) at the Institute of Biotechnology, University of Helsinki, Finland. The amplified fragment lengths were assigned to allelic sizes at the accuracy of one base pair with Peak Scanner version 1 software (Applied Biosystems).

### 2.3 Statistical methods

A chloroplast multilocus haplotype was combined for each individual based on cpSSR alleles in the data set, excluding individuals with missing data (*N* = 588). The number of multilocus haplotypes (MLG, multilocus genotypes) and the number of expected haplotypes (eMLG, expected multilocus genotypes at the smallest sample size ≥10 based on the rarefaction) were computed with R 3.3.2 (R Core Team, 2016) package *poppr *(Kamvar, Brooks, & Grünwald, 2015; Kamvar, Tabima, & Grünwald, 2014).

To characterize genetic variation based on chloroplast cpSSR loci, allelic diversity (*N* = 603) and number of multilocus haplotypes (*N* = 588, without missing data) were calculated over the entire sample set, for each region (*n* = 8) and population (*n* = 27), for each of the six clusters identified by BAPS (*K* = 6; see below), and for both endophyte infections statuses (*N* = 566, infected E+ and uninfected E−). We also calculated these statistics separately for plants with different ploidy levels (*N* = 566, 2n = 28, 42, 56). The percentage of polymorphic loci (%P), the mean number of observed alleles over loci (Na), the mean effective number of alleles over loci (*N*_e_), the mean number of unique alleles over loci (*N*_p_), and the unbiased haploid genetic diversity over loci (uh) were computed using GenAlEx 6.5 software (Peakall & Smouse, 2006, 2012). We also tested for significance of differences in allelic diversity estimates (*N*_e_, uh) of the loci (altogether 13 cpSSR loci) between regions, BAPS clusters, and endophyte infection status groups (infected E+/uninfected E−) in four regions (Faroe Islands, Iceland, Kevo, Spain) with sufficient number of E+ plants (see Table 1). These tests were conducted by employing related samples nonparametric Friedman's two‐way analysis of variance by ranks or related samples Wilcoxon signed rank nonparametric test for two groups (Sokal & Rohlf, 1995; Wilcoxon, 1945), arranged for paired observations using IBM SPSS Statistics for Windows, version 23 (IBM Corp., Armonk, NY, USA; IBM Corp, 2015).

**Table 1:** Allelic diversity estimates and numbers of unique haplotypes in *Festuca rubra* across eight regions and six BAPS clusters based on 13 cpSSR loci; BAPS clusters: S1 = *Spain1‐group, S2 = Spain2‐group, F = Fennoscandia‐group, A = Atlantic‐group*

	Allelic diversity					Number of multilocus haplotypes		
	*N*	%P	*N*_e_	*N*_p_	uh	*N*	MLG	eMLG
*By region*								
Faroe Islands	130	100	1.7	1.5	0.361	124	65	18.8
Greenland	43	61.5	1.1	0.1	0.078	43	9	7.0
Southern Finland, Hanko	63	100	1.6	0.4	0.291	63	30	16.2
Iceland	56	100	1.4	1.2	0.279	55	22	13.4
Northern Finland, Kevo,	147	100	1.4	1.0	0.279	146	55	14.6
Northern Finland, Kilpisjärvi	55	76.9	1.4	0.6	0.219	54	18	11.0
Southern Norway, Bergen	29	76.9	1.6	0.2	0.314	29	12	12.0
Spain	80	100	2.6	1.2	0.538	74	58	25.5
*By BAPS cluster*								
*F*	239	100	1.3	0.5	0.130	238	59	12.4
*A*	187	84.6	1.1	0.4	0.103	184	44	10.1
*S1*	54	100	2.3	0.9	0.470	52	43	20.9
*S2*	70	100	2.4	0.8	0.475	66	52	21.5
*A–F*	29	100	3.5	3.2	0.625	24	24	24.0
*F–A*	24	100	2.7	1.2	0.616	24	16	16.0
Total	603	100	1.9	‐	0.408	588	238	20.6

Note

Population and sample information are given in Supporting information Appendix S1.

*N*, sample size; %P, percentage of polymorphic loci; *N*_e_, mean effective number of alleles over all loci; *N*_p_, mean number of private alleles over all loci; uh, unbiased haploid genetic diversity over all loci; MLG, number of unique multilocus haplotypes; eMLG, number of estimated unique multilocus haplotypes.

The genetic relationships of individuals were assessed using a model‐based approach using the Bayesian Analysis of Population Structure (BAPS) software, version 6.0 (Corander, Cheng, Marttinen, Sirén, & Tang, 2013; Corander & Tang, 2007), by applying a nonspatial genetic mixture analysis with known populations to the cpSSRs results of the whole sample set, with the clustering of linked loci option (Corander & Tang, 2007). Allele frequencies and the number of genetically diverged groups in a population were treated as random variables. To determine the most probable number of genetically diverged clusters (*K*), the genetic mixture analysis at the individual level was conducted by performing 100 iterations of *K* (from 2–35). Under default settings, BAPS identified that the optimal partition of populations into clusters would be obtained with 20 clusters (*K* = 20; the highest marginal log‐likelihood value = −4,514.8). A UPGMA tree was constructed based on the Kullback–Leibler divergence matrix, provided as an output of the BAPS analysis. However, as observed by, for example, Rodriquez et al. (2013), BAPS has a tendency to overestimate the number of small clusters as the most likely number of K. Therefore, we used a Fixed K model option, based on the K numbers in the uppermost hierarchical levels of the genetic structure shown in the UPGMA tree of *K* = 20 (Supporting information Appendix S2), and carried out a Fixed K model analysis with 100 runs of iterations for *K* = 3 and *K* = 6. The UPGMA trees were constructed, as described above. An admixture analysis of individuals was conducted with BAPS, based on the mixture clustering with *K* = 6 with 200 simulations using posterior allele frequencies. Based on the admixture results, the Plot Gene Flow function of the BAPS software was used to estimate and illustrate a network of clusters at *K* = 6. In addition, we also conducted a principal coordinate analysis (PCoA) in the GenAlEx v6.5 software; principal coordinates determined for the pairwise individual‐by‐individual haploid genetic distance matrix to reveal genetic similarities among individuals in different regions.

A hierarchical analysis of molecular variance (AMOVA) was used to estimate the degree of genetic differentiation among regions, among populations, and among the six clusters (*K* = 6) obtained by BAPS program by using Arlequin software version 3.5 (Excoffier & Lischer, 2010), which estimates genetic structure indices using information on the allelic content of haplotypes and allele frequencies (Excoffier, Smouse, & Quattro, 1992). Pairwise differentiation among regions, populations, the six BAPS clusters (K = 6), and between endophyte infection status groups (infected E+/uninfected E−) in four regions (Faroe Islands, Iceland, Kevo, Spain) with sufficient number of E+ plants was estimated as pairwise *F*_ST_ values (Weir & Cockerham, 1984) by using Arlequin software version 3.5 (Excoffier & Lischer, 2010). F_ST_ is unbiased with respect to sample size, and it adjusts allele frequency estimates with respect to sample sizes (Weir & Cockerham, 1984; see Whitlock, 2011). The significance of the fixation indices was computed with 10,100 nonparametric permutations.

## 3 RESULTS

### 3.1 Allelic diversities and numbers of multilocus haplotypes

A total of 238 cpDNA multilocus haplotypes (MLG) were identified in the sample set (*N* = 588) based on the 13 combined cpDNA microsatellite loci. Among regions, the different haplotype numbers in relation to sample sizes (eMLG) and allelic diversity estimates (*N*_e_ and uh) were clearly highest in Spain and lowest in Greenland, but relatively high genetic diversities were also observed in the Faroe Islands (Table 1). The allelic diversity estimates differed significantly among regions (*N* = 8; *N*_e_, *p* < 0.001; uh, *p* < 0.001). The highest mean numbers of private alleles over all loci (*N*_p_) were observed in the Faroe Islands, Iceland, and Spain (1.2–1.5), and lowest (0.1–0.4) in Greenland and southern Fennoscandia in Hanko and Norway (Table 1).

Among populations, haplotype numbers, allelic diversity estimates, and mean numbers of private alleles over loci varied largely within regions, except in Spain and Greenland (Supporting information Appendix S1). Highest allelic diversity (*N*_e_: 2.1–2.8 and uh: 0.456–0.608) and number of haplotypes (eMLG: 10–12/population) were detected in the Spanish populations, in FAS2 and FAS5 from the Faroe Islands and in HA2 from southern Fennoscandia. Lowest allelic diversity (*N*_e_: 1–1.1 and uh: 0.040–0.106) and numbers of different haplotypes in relation to sample sizes (eMLG = 3.2–5/population) were found in three populations in northern Fennoscandia (RIS1, RIS2, MS2K), in both Greenlandic populations, and in Iceland (IC1). The highest mean numbers of private alleles (*N*_p_ = 0.3–0.5) were observed in southern populations in Spain (SPLV, SPPOR) and northwestern populations in the Faroe Islands (FAS1, FAS2, FAS5), Iceland (IC1, IC2) and also in one population (RBS1) in northern Fennoscandia.

### 3.2 Population structure

Genetic relationships among individuals within regions were first investigated by PCoA (Supporting information Appendix S3). The first, second, and third principal coordinates explained 24.4%, 15.0%, and 4.9%, respectively, altogether 44.3% of the variability. No clear main groups were identified by this analysis, as samples from different regions were located on different overlapping parts of the plot.

Based on the fixed K model produced by BAPS at *K* = 6, individuals were distributed in four relatively large clusters (*clusters A, F, S1, and S2*) and two small clusters (*clusters A–F *and *F–A*; the highest marginal log‐likelihood value = −4,856.64; Figure 2; Supporting information Appendices S1 and S4). *Cluster A* (*n* = 187; “*Atlantic‐group*”) included mainly northern Atlantic individuals from the Faroe Islands, Iceland, and Greenland. *Cluster F* (*n* = 239; “*Fennoscandia‐group*”) was mainly characterized by Fennoscandian individuals from southern and northern Finland and Norway. *Cluster S1* (*n* = 54; “*Spain1‐group*”) included individuals from Spain (53.7%), as well as some from southern Fennoscandia and northern Atlantic. C*luster S2* (*n* = 70; “*Spain2‐group*”) included individuals mostly from Spain (71.4%), but also from the northern Atlantic and southern Fennoscandia. The remaining individuals from northern Fennoscandia and Atlantic area were included in the smallest *clusters F–A* and *A–F*.

The AMOVA analysis showed that 50.9% of genetic variation lies among clusters and 49.1% within clusters (*df *= 5, v.c = 1.56, *p* < 0.001; *df *= 1,200, v.c 1.51, *p* < 0.001, respectively), which indicates a very high degree of genetic differentiation among clusters. Based on pairwise *F*_ST_ values between clusters, all cluster pairs were significantly differentiated from each other, but differentiation was highest between *Atlantic‐group *(*A*) and the other clusters, and *Fennoscandia‐group* (*F*) and the other clusters, and lowest between *Spain1‐ and Spain2‐groups*, which co‐occurred abundantly in the same populations (Supporting information Appendix S5).

Significant differences were detected in the allelic diversity estimates (*N*_e_, uh) among the six clusters (Table 1). The largest clusters *Atlantic‐group *and *Fennoscandia‐group* possessed clearly the lowest levels of allelic diversity and numbers of different haplotypes, whereas considerably higher levels of allelic diversity and numbers of different haplotypes were found in both *Spain‐groups*. However, the highest levels of genetic diversity and private allele numbers were found in the smallest *clusters A–F *and *F–A*.

**Figure 3:**
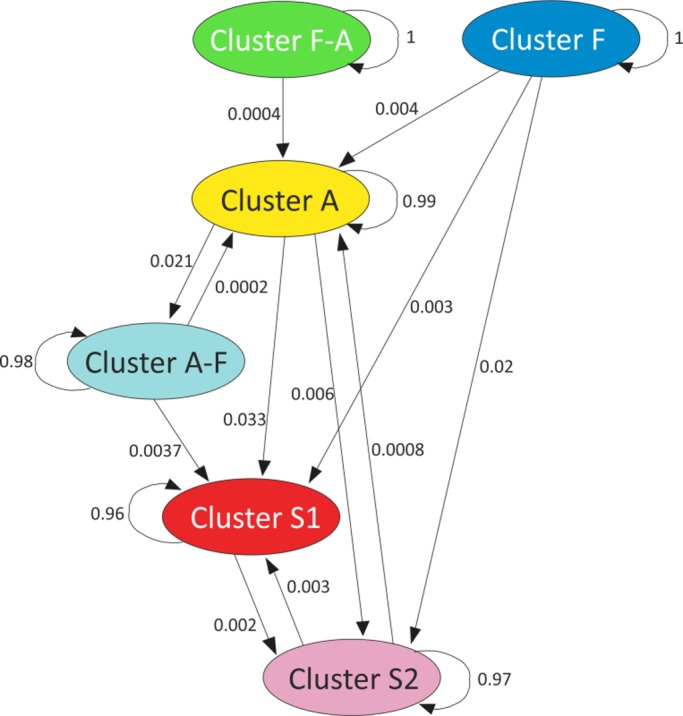
A gene flow network identified for the six clusters (*K* = 6) as obtained by BAPS in *Festuca rubra*. Gene flow is shown by weighted arrows, which indicate relative average amounts of ancestry coming from the source cluster but present now among individuals assigned to the target cluster. Cluster S1 = *Spain1‐group*; cluster S2 = *Spain2‐group*; cluster *F* = *Fennoscandia‐group*. Estimated ancestral admixture (gene flow) is indicated by weighted arrows (Tang, Hanage, Fraser, & Corander, 2009)

The results of the admixture analysis shown as a gene flow network of six clusters (*K* = 6) are summarized in Figure 3. Inter‐cluster ancestral gene flow varied from 0.02% to 3.3% (Figure 2). *Fennoscandia‐group* and *cluster F–A* with 66.7% of a Fennoscandian gene pool showed no ancestral gene flow from the other clusters. *Atlantic‐group* had 0.4% to 0.08% of admixed effect other clusters. *Spain1* *‐group *had some ancestral gene flow from the *Atlantic‐group* (3.3%). On the contrary, *Spain2‐group *had its main source of gene flow from *Fennoscandia‐group* (2.0%).

### 3.3 Genetic differentiation between regions and populations

The AMOVA analysis showed that 23.5% of genetic variation lies among regions, 10.6% among populations within regions and the remaining two‐thirds (65.9%) within populations (Table 2), indicating moderate genetic structure. The pairwise *F*_ST_ values among all populations are shown in Supporting information Appendix S6.

**Table 2:** Results of AMOVA analysis within the whole data set (*n* = 603), and separately within (a) Spain, (b) North Atlantic, and (c) Fennoscandia

Origin	*df*	SS	Variance components	Variance (%)	*p*
Whole data set					
Among regions	7	761.6	0.65	23.51	<0.001
Among populations within regions	19	278.6	0.29	10.58	<0.001
Within populations	1,179	2,136.7	1.81	65.91	<0.001
(a) Spain					
Among populations	2	28.9	0.21	6.01	0.003
Within populations	157	515.1	3.28	93.99	
(b) North Atlantic					
Among regions	2	29.4	0.03	1.49	0.199
Among populations within regions	8	86.6	0.22	11.37	<0.001
Within populations	447	754.4	1.69	87.14	<0.001
(c) Fennoscandia					
Among regions	3	95.1	0.10	4.99	0.052
Among populations within regions	9	163.1	0.37	18.83	<0.001
Within populations	575	867.2	1.51	76.18	<0.001

**Figure 4:**
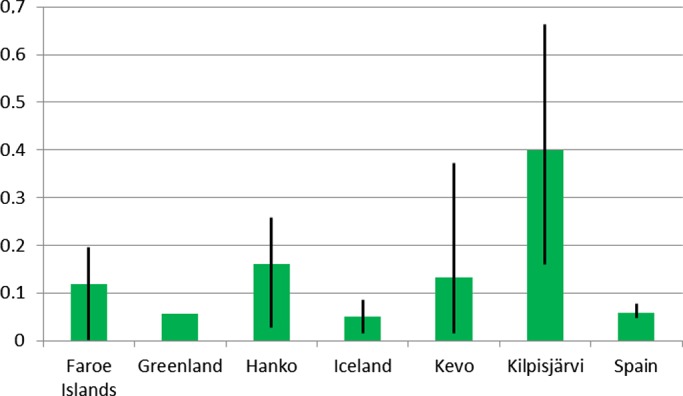
Average pairwise *F*_ST_ values among populations within regions in *Festuca rubra*. The black lines show the range of exact *F*_ST_ values. The pairwise *F*_ST_ values among all the populations are shown in Supporting information Appendix S6. Population and sample information are given in Supporting information Appendix S1

Within Spain, low levels of genetic differentiation were detected among populations (6%) and the majority (94%) of the variation lies within populations (Table 2). Pairwise *F*_ST_ revealed significant differentiation only between SPGD and SPLV populations (*F*_ST_ = 0.077, *p* = 0.003), but no differences between SPGD and SPPOR, and SPLV and SPPOR populations suggesting effective gene flow between most Spanish populations (Figure 4; Supporting information Appendix S6).

In the northern Atlantic area, no significant genetic differentiation was detected among the three islands, which indicates effective gene flow among the Faroe Islands, Iceland, and Greenland (Table 2). Instead, significant differentiation was found among populations within the Faroe Islands (*F*_ST_ = 0.125, *p* < 0.001), while no differentiation was detected among populations in Iceland (*F*_ST_ = 0.054, *p* = 0.086) or Greenland (*F*_ST_ = 0.057, *p* = 0.132; Figure 4).

In Fennoscandia, no genetic differentiation was found among four regions (Hanko, Norway, Kilpisjärvi, Kevo), but clearly more genetic differentiation was located among populations within regions. This indicates that the Fennoscandian regions are more differentiated from each other than regions in the northern Atlantic area (Table 2). Moderate genetic differentiation was observed among populations in Kevo (*F*_ST_ = 0.160, *p* < 0.001) and Hanko (*F*_ST_ = 0.190, *p* < 0.001), and greater among populations in Kilpisjärvi (*F*_ST_ = 0.389, *p* < 0.001).

### 3.4 Genetic structure and endophyte infection rates

The associations of the infection rates and allelic diversity estimates of populations differ based on the geographic locations. In the Faroe Islands, the allelic diversity estimates (*N*_e_, uh) were significantly greater among infected than among uninfected plants (*N*_e_, *p* = 0.001, uh, *p* = 0.001), while more genetic diversity was found among uninfected plants in Iceland and Kevo (*N*_e_, *p* = 0.001, *p* = 0.009; uh, *p* = 0.001, *p* = 0.011, respectively). In contrast, allelic diversity estimates did not differ between infected and uninfected plants in Spain (Table 3; *N*_e_, *p* = 0.552, uh, *p* = 0.345).

**Table 3:** Allelic diversity estimates and numbers of unique haplotypes among the plants of *Festuca rubra* with *Epichloë festucae* infection (E+) or without infection (E−) in different geographical regions based on 13 cpSSR loci

	Faroe Islands		Iceland		Finland, Kevo		Spain	
	E+	E−	E+	E−	E+	E−	E+	E−
Allelic diversity								
*N*	67	54	21	34	98	36	66	14
%P	100	100	84.6	100	100	92.3	100	92.2
*N*_e_	1.724	1.558	1.272	1.462	1.277	1.598	2.634	2.499
*N*_p_	0.846	0.462	0.154	0.923	0.615	0.231	1.000	0.077
uh	0.378	0.325	0.206	0.302	0.210	0.352	0.536	0.558
Haplotype diversity								
*N*	66	51	20	34	98	36	61	13
MLG	39	28	9	15	40	16	51	12
eMLG	9.9	8.6	6.2	7.5	6.9	7.9	12.3	12

Notes

Population and sample information are given in Supporting information Appendix S1.

*N*, sample size; %P, percentage of polymorphic loci; *N*_e_, mean effective number of alleles over all loci; *N*_p_, mean number of private alleles over all loci; uh, unbiased haploid genetic diversity over all loci; MLG, number of unique multilocus haplotypes; eMLG, number of estimated unique multilocus haplotypes.

The pairwise *F*_ST_ values showed significant genetic differentiation between infected and uninfected plants in Kevo (*n* = 134; *F*_ST_ = 0.045, *p* = 0.012), but no differentiation between the plant groups in the Faroe Islands, Iceland, and Spain (*n* = 121, *F*_ST_ = 0.020, *p* = 0.066; *n* = 55, *F*_ST_ = 0.004, *p* = 0.830; *n* = 80, *F*_ST_ = 0.015, *p* = 0.587, respectively).

Infection frequencies varied from 35.9% to 73.9% among the BAPS clusters (*K* = 6, Supporting information Appendix S7), being highest in *Spain1‐ and 2‐groups *and lowest in *Atlantic‐group*.

### 3.5 Genetic structure and ploidy levels

Among the six clusters obtained by BAPS (*K* = 6), all three ploidy levels (2n = 28, 42, 56) occurred in *Atlantic‐, Fennoscandia‐,* and *Spain1*‐*groups *and also in the small *cluster A‐F *(Supporting information Appendices S7 and S8). Hexaploids predominated in all other clusters, except for *Spain1‐ and Spain2‐groups*, which were largely characterized by the presence of tetraploids. Tetraploids were extremely rare among *Atlantic‐ *and *Fennoscandia‐groups*, but more common in the small *cluster A–F*. Octoploids were present in all other clusters at low frequencies, except in the *Spain1‐group *and in the small northern *cluster F–A*.

## 4 DISCUSSION

### 4.1 Population genetic structure and geographic differentiation of *F. rubra*

According to our cpDNA data, about 40% of the sampled plants had unique haplotypes and the total genetic diversity was relatively high in the studied natural populations of *F. rubra*. Similarly, high levels of genetic diversity have been revealed by cpDNA markers across Europe in *Lolium perenne* (McGrath, Hodkinson, & Barth, 2007), which is widely used as a cultivated agricultural species. McGrath et al. (2007) suggested that migration of seed material by natural and anthropogenic means, including gene flow from cultivars, can contribute to a high level of variability.

In *F. rubra,* the amount of genetic variation was highest in Spain near a potential area of a glacial refugium, and a clear reduction in genetic diversity was detected in previously glaciated areas of the northern Atlantic region and Fennoscandia. Moreover, nearly all haplotypes from Spain fell in *Spanish‐groups* indicating clear geographic clustering in the study area. The high amount of genetic diversity of *F. rubra* occurring in Spain compared to other regions indicates the possibility of glacial refugia in the Iberian Peninsula, similarly as it has been found for several other plant and animal species in Europe (Fjellheim, Rognli, Fosnes, & Brochmann, 2006; Hewitt, 1996, 1999, 2000, 2004; Keppel et al., 2012). The gene flow plot produced by BAPS and low levels of differentiation among the Spanish populations suggest that the gene pools of *Spain 1‐ and 2‐groups* have intermixed and also received genetic make‐up from either Atlantic‐ or Fennoscandia‐group to different degrees. This result indicates that multiple maternal lineages have persisted near the Iberian Peninsula through climatic oscillations. Evidently, *Spain 1‐ and 2‐groups* had contact zones with *F. rubra* both in northern Atlantic and southern Fennoscandia, thus suggesting effective colonization via seed dispersal from the Iberian Peninsula toward formerly glaciated areas in the northern Europe.

Most haplotypes originating from the Northern Atlantic islands fell into the highly differentiated *Atlantic‐group*, which showed decreasing amounts of genetic diversity toward north. Rapid range expansion from refugia to previously glaciated northern areas has often been found to be associated with a decreased genetic diversity in the north (Eidesen et al., 2013; Hewitt, 1999). The extremely low level of differentiation among populations in the northern Atlantic islands confirms the presence of effective postglacial long‐distance seed dispersal events among the Faroe Islands, Iceland, and western Greenland. Previously, Schönswetter, Suda, Popp, Weiss‐Schneeweiss, and Brochmann (2007) have detected comparable extensive dispersal over enormous distances among three genetic subgroups of *Juncus biglumis*, all showing circumarctic distribution areas with largely overlapping distribution patterns. Trans‐Atlantic dispersal has recently been proposed for other grasses and plants (Brochmann et al., 2003; Jimenez‐Mejías et al., 2012; Schönswetter, Elven, & Brochmann, 2008; Westergaard, Alsos, Engelskjøn, Flatberg, & Brochmann, 2011), although the Atlantic ocean has also been seen as an impenetrable barrier to seed dispersal (Eidesen et al., 2013; Hultén, 1958). Interestingly, even though plants from eastern and western Greenland have often been found to be genetically different as a result of the Greenlandic ice cap barrier for gene flow (Alsos, Torbjorn, Normand, & Brochmann, 2009; Eidesen et al., 2013), our cpDNA data show that seed dispersal has occurred between western Greenland and northern Atlantic islands. The extremely low genetic diversity level observed in Greenland is likely due to founder effect and genetic drift and very rare occurrences of colonizations to the island. However, plant dispersal from ice free areas in eastern or western Greenland has been suggested to be considerable (Eidesen et al., 2013). Yet, based on our results on *F. rubra,* this is unlikely, since almost no private alleles and unique haplotypes were found in the Greenlandic populations. It is also possible that long‐distance dispersal events have occurred from northern America, which can be resolved in future studies with circumpolar sampling.

The Faroe Islands and Iceland have received migrants from all genetic groups, indicating that the area is a contact zone of seed material originating from southern and northern European maternal lineages. Similarly in *Carex bigelowii,* the contact zones of different lineages have been identified to occur in Iceland, where most haplotypes belong to the European genetic cluster, including samples from Central Europe, Scandinavia, and UK (Schönswetter et al., 2008). In *F. rubra*, based on the gene flow plot obtained by BAPS, the *Atlantic‐group* has received genetic material in small quantities from the *Fennoscandia‐group* and *Spain‐2‐group*, which suggests that the glacial origin of the *Atlantic‐group* might be in some southern European peninsula, similarly as detected in *C. bigelowii* (Schönswetter et al., 2008) and *Ranunculus glacialis* L. (Schönswetter, Paun, Tribsch, & Niklfeld, 2003), but further sampling from different potential refugial areas is needed to confirm that. The accumulation of different genetic groups in the northern Atlantic islands may be a consequence of natural long‐distance seed dispersal from other sources not studied here or a consequence of anthropogenic seed dispersal through seed trade and movement of farm animals and cattle feed between the continent and islands (see Linder et al., 2018). Human‐induced seed dispersal is also suggested to be a substantial factor to shape the population genetic structure of forage and turf grass *L. perenne* in Europe (McGrath et al., 2007). Moreover, the occurrence of the distinct and genetically diverse cluster A‐F that contributes to increased genetic diversity in the Faroe Islands and Iceland may indicate periglacial survival in ice free areas in the Faroe Islands, as suggested in many studies (Brochmann et al., 2003; Jimenez‐Mejías et al., 2012; Westergaard, Alsos, Popp et al., 2011).

The majority of haplotypes in southern and northern populations in Fennoscandia fell into the highly differentiated *Fennoscandia‐group*, which shows, like the *Atlantic‐group*, considerably lower genetic diversities compared to *Spain‐groups*. The increased genetic diversity in the southern Fennoscandian populations may be due to postglacial range expansion of both Spanish‐groups forming contact zones in the area, as discussed earlier. In northern Finland, the majority of *F. rubra* populations consist almost merely of haplotypes from *Fennoscandia‐group *and *F‐A cluster,* but also migrants from the *Atlantic‐group* and *A‐F cluster* are present at small frequencies in some northern populations, indicating long‐distance seed dispersal from the northern Atlantic islands to northern Finland. Eidesen et al. (2013) have found strong connectivity among Scandinavia, Iceland, and the British Isles in a multispecies analysis of spatial genetic structures in northern species. Moreover, several studies have confirmed that northern Europe was colonized not only from the south, but was heavily influenced by the postglacial immigration from the east, thus forming contact zones of individuals with different lineages, which increased the genetic diversity of populations (Eidesen et al., 2013; Fjellheim et al., 2006; Jimenez‐Mejías et al., 2012; Malm & Prentice, 2005; Skrede, Eidesen, Portela, & Brochmann, 2006; Westergaard, Alsos, Engelskjøn et al., 2011). In *F. rubra*, based on the gene flow plot obtained by BAPS, the *Fennoscandia‐group* showed no gene flow from the other clusters studied here, thus indicating their origin being from another refugial area than those studied here, perhaps in Italy, the Balkans or even Beringia as a referee suggested.

In our data set, the highest levels of differentiation were found in the four northernmost populations situated in the subarctic. Extremely low levels of genetic diversity and haplotype numbers observed in two populations in Kilpisjärvi (RIS1, RIS2) give an indication of small founder populations, which have encountered severe population bottlenecks and genetic drift. This may also be due to the predominance of vegetative reproduction in those populations (Wäli et al., 2007; Henry Väre, personal observations). However, polyploid arctic plants can maintain their genetic variation in spite of bottlenecks, because individual plants are able to retain most of the population's gene pool in the form of fixed heterozygosity (Brochmann et al., 2004). Such effect might be stronger in the RIS2 population with a predominance of octoploids than in RIS1 with a prevalence of hexaploids. Similarly, among other arctic polyploid plants, most genetic variation has been found as fixed heterozygosity and as variation among different populations (Brochmann et al., 2004). In contrast, the subarctic populations RBS3 and RIS3 were found to be diverged from all other populations because of abundant occurrences of rare and genetically diverse haplotypes of *F‐A cluster*, which has increased the amount of genetic diversity and numbers of unique haplotypes. One explanation for the genetic distinctiveness of the *F‐A cluster* is the glacial survival in nunataks or in other in situ glacial refugia in Norway (Eidesen et al., 2013; Brochmann et al., 2003, and references therein). There is fossil and molecular evidence that supports the idea of refugia in Scandinavia for *Sagina caespitosa* (Westergaard, Alsos, Popp et al., 2011) and conifer trees (Parducci et al., 2012). However, representatives of the *F‐A cluster* can be found at low frequencies also in the Atlantic islands, which indicate long‐distance seed dispersal among the northern areas. Thus, the origin of this rare cluster remains unclear. Moreover, we cannot exclude the possibility of an anthropogenic origin for *F-A cluster* haplotypes, as cultivar haplotypes might have dispersed to natural habitats in northern Finland from seed mixtures sown along roadsides (Wäli et al., 2007).

### 4.2 *E. festucae* infection rates versus population genetic structures

In *F. rubra*, the infection rates of *E. festucae* in relation to genetic variability of the host populations varied among geographical areas, which may due to differences in population histories and possible costs of systemic fungi in varying environmental conditions. Gundel et al. (2010) have proposed that stable intermediate levels of genetic variability are beneficial for the host plant's fitness and effectiveness of mutualism. Low levels of genetic variability among infected plants can cause a reduction in the host plant's fitness, especially in harsh conditions, while very high levels of genetic variability among infected plants should show similar or lower fitness compared to uninfected plants due to changes in compatibility and out‐breeding depression (Gundel et al., 2010). Similar levels of genetic variability with no differentiation between the E+ and E− plants in Spain suggest that individual plants have common historical origins despite differing endophyte status in the region. Similarly, no differences have been found in genetic diversities among endophyte‐infected and uninfected plants in *Festuca eskia* populations in the Pyrenees in southern Europe, near suggested refugium areas (Gonzalo‐Turpin et al., 2009). Similar local patterns of genetic diversities among infection groups can be promoted by several underlying processes, for example, the production of both infected and noninfected offspring by the same infected plants. *E. festucae *infections can be lost in seedlings due to failures in the colonization of systemic fungi in plant seeds or vegetative reproductive parts in plants descending from endophyte‐infected maternal families (Saikkonen et al., 2010, 2004). On the other hand, occasional horizontal transmission by sexual fungal spores can affect genetic diversity patterns by increasing infection rates in previously uninfected plants. In the Spanish populations of *E. festucae*, the occurrence of fertile stromata has been detected occasionally in *F. rubra, *but more frequently in other grass species (Zabalgogeazcoa et al., 1999). Moreover, effective gene flow among Spanish populations and *Spain1 and 2‐groups*, and the long‐lasting history of the Spanish populations near refugia have potentially influenced the genetic mixing of E+ and E− plants in the region.

In contrast, higher levels of genetic diversity and significant differentiation were found among uninfected than infected plants in Kevo in northern Finland. This may be due to a selective disadvantage caused by the fungus under a short growing season and harsh environmental conditions (Ahlholm et al., 2002; Wäli et al., 2007; Leinonen et al., in prep.), which would lead to an increase in the number of uninfected plant genotypes in subarctic conditions. Leinonen et al. (*in prep*) observed in a transplantation experiment in Kevo that infected plants originating from the Faroe Islands had a lower fitness compared to uninfected plants. This result suggests that long‐distance seed dispersers of *F. rubra* may lose infections during the establishment process, especially in harsh conditions. The genetic mismatches between the plant and the fungus genotypes may play a role in infection losses of new plant genotypes during the establishment process (see Saikkonen et al., 2010, 2004). This view is supported by an observation that only little genetic variation is found in the subarctic *E. festucae* populations in Kevo, thus indicating that only few fungus genotypes are mainly vertically transmitted from maternal plants to offspring in the region (Wäli et al., 2007; see also Sullivan & Faeth, 2004; Zhang, Ren, Ci, & Gao, 2010). Moreover, wind dispersed pollen is likely to generate new genotypes in the populations of *F. rubra*, causing asymmetric rates of gene flow and interactions of incompatible genotypes, as suggested for *Neotyphodium *and its host *Festuca arizona* by Sullivan and Faeth (2004). Lower diversities of symbiotic fungi compared to the host diversities have been observed in the natural populations of *Neotyphodium* species and their host *Achnatherum*, which indicates more restricted gene flow distances in fungi than in their plant hosts (Zhang et al., 2010). Seeds produced by outcrossing of genetically distant parents can cause incompatibilities between the fungus and the grass host, and consequently, the vertical transmission of the fungus with seeds would fail (Gundel et al., 2010; Saikkonen et al., 2010). In the *F. rubra *system, genetic incompatibilities between hosts and *E. festucae* may increase the accumulation of uninfected new genotypes in populations, followed by greater genetic differentiation between E+ and E− plants, as we observed in Kevo. Contrary to Kevo and Iceland, more genetic diversity was present among infected than uninfected plants in the Faroe Islands, and no genetic differentiation was observed between the plant groups. In this region, several different genetic groups occur frequently, which has possibly enhanced the maintenance of infections during the establishment process and, consequently, resulted in the production of several genetically different maternal lineages of both plants and fungi.

Very low numbers or no *E. festucae* infections were observed among the plants in Greenland and southern Fennoscandia and also in one region (Kilpisjärvi) in northern Fennoscandia. Based on the hypothesis by Gundel et al. (2010), possible losses of infections in populations located in southern Fennoscandia may be caused by genetic mismatches between the host and fungus, since, in that region, two genetically highly distant genetic groups, *Fennoscandia‐* and S*panish‐groups* occur abundantly (see also Saikkonen et al., 2010, 2004). In contrast in the populations in Kilpisjärvi (RIS1, RIS2) and Greenland, the possible losses of infections may be due to the observed extremely low genetic variability among the plants causing material or energetic costs to maintain endophytes under stressful environmental conditions when seed production can be lost by fitness‐depressed host plants (Gundel et al., 2010). Nevertheless, lack of infections can also be caused by introductions of noninfected seed material into populations without fungal gene flow. Interestingly, only one infected plant was observed in Greenland, the haplotype belonging to *Spain2‐group*, whereas all other haplotypes belonged to genetically very distant *Atlantic‐group, *which may indicate its recent introduction.

### 4.3 Ploidy levels versus population genetic structures

In *F. rubra,* the frequencies of plants with higher ploidy levels were clearly greater in *Atlantic‐* and *Fennoscandia‐groups* than in *Spain‐groups,* which may suggest the selective advantage of higher ploidy levels in the genetically poorer populations in the north. Sampoux and Huyghe, (2009) have proposed for the fine leaved fescue lineages that cytotypes with high ploidy levels might have been efficient colonizers and competitors. Therefore, such lineages would have expanded more effectively in the northern areas. Polyploidy can facilitate a species’ competitive ability and ability to colonize novel environments. They may have a broader ecological range than their diploid relatives, especially in northern latitudes (Brochmann et al., 2004; Linder & Barker, 2014). In *F. rubra,* almost all distinct ploidy levels (tetra‐, hexa‐ and/or octoploids) occur among *Atlantic‐, Fennoscandia*‐, and *Spain1‐ *and *Spain2‐groups*, which shows that plants of different ploidy levels are closely related which suggests independent formation of polyploids in different maternal lineages in different geographic areas. Similarly in *Dupontia *(Poaceae), several different ploidy levels were detected in three diverged genetic groups in a circumarctic area (Brysting, Fay, Leitch, & Aiken, 2004).

## 5 CONCLUSIONS

This is the first comprehensive study that illustrates the genetic structure and geographic differentiation over a wide area in natural populations of *F. rubra* differing in the infection rate of the symbiotic fungus *E. festucae* and ploidy level. It shows that the level of genetic variation in different geographic regions is evidently highly dependent on the postglacial colonization history. The relationship between the host and the symbiotic fungus in the sense of genetic variability is not consistent but appears to differ among geographic regions. Diverse genetic structure among regions in *F. rubra* gives valuable background information for the development of *F. rubra* cultivars that would possess suitable combinations of the host and systemic fungus genotypes for agricultural use affected by abiotic stresses like drought, salinity, and high temperature. The present study is part of a larger ongoing project investigating local adaptation and phenotypic variation in European *F. rubra* and its symbiotic fungus *E. festucae*.

## CONFLICT OF INTEREST

None declared.

## AUTHOR CONTRIBUTIONS

H.V., M.H., and K.S. designed the collection of the data and performed sampling. M.v.C. and H.K. designed the genetic study. M.v.C. genotyped the samples. M.v.C. and P.L. analyzed the data. M.v.C. wrote the manuscript with contributions by all authors.

## Data Availability

Data (population names, endophyte infection status, ploidy levels, and microsatellite genotypes of the individuals) are archived as Supporting information on the Ecology and Evolution web site (Supporting information Appendix S9).
