# Supplementary material for: Postglacial colonization history reflects in the genetic structure of natural populations of Festuca rubra in Europe
Source: Ecol Evol. 2019 Mar 4;9(6):3661–74. doi: 10.1002/ece3.4997 (PMC6434542; doi:10.1002/ece3.4997)
Supplement: Supplementary file 1 [file ECE3-9-3661-s001.docx]

Supporting Information Appendices

Supporting information Appendix S1.

The information of geographic locations, taxonomic identities, ploidy levels, endophyte infection rates, allelic and haplotype diversities, and numbers of individuals per BAPS clusters at K=3 and K=6 in 27 studied populations located in Spain and northern Europe.

Supporting information Appendix S2.

A UPGMA tree was constructed based on the Kullback-Leibler divergence matrix, provided as an output of the analysis of the software the Bayesian Analysis of Population Structure (BAPS) when applying a non-spatial genetic mixture analysis with known populations to the cpSSRs results of the whole sample set (n=603), with the clustering of linked loci option (Corander & Tang, 2007). BAPS identified that the optimal partition of populations of *Festuca rubra* into clusters would be obtained with 20 clusters.

Supporting information Appendix S3.

Genetic relationships among *Festuca rubra* individuals (n=603) within eight studied regions as determined by principal coordinates analysis.


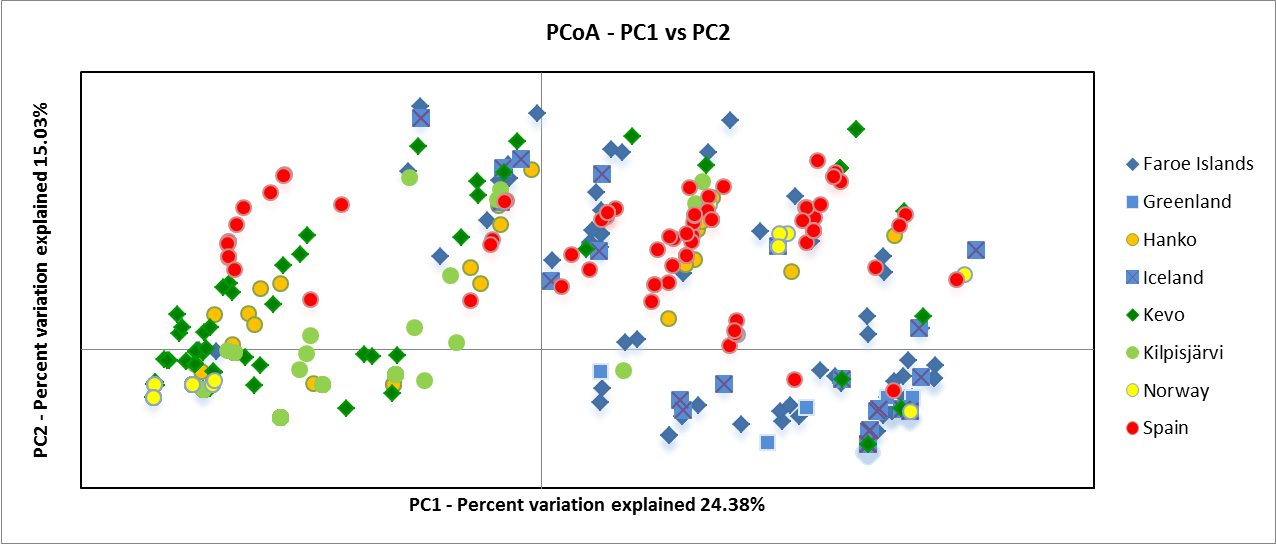


Supporting information Appendix S4.

The Bayesian Analysis of Population Structure (BAPS) cluster assignments A) at K=3 and at K=6 for cpSSR data of 603 wild European individuals of Festuca rubra. B) UPGMA trees at K=3 (clusters A, F and S) and at K=6 (clusters: A=*Atlantic-group*, F=*Fennoscandia-group*, S1= *Spain1-group*, S2= *Spain2-group*, *F-A-cluster, A-F-cluster*) based on the Kullback-Leibler divergence matrices, as obtained by BAPS analysis.

**
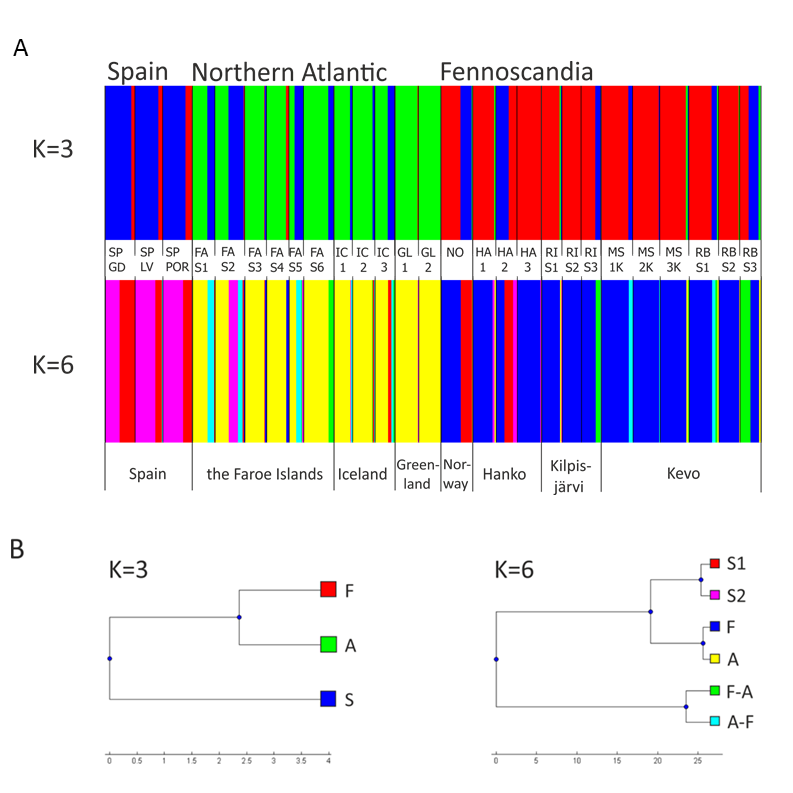
**

Supporting information Appendix S5.

Genetic differentiation based on pairwise F_ST_ values among six clusters (K=6) obtained by BAPS in *Festuca rubra*. Clusters: F*=Fennoscandia-group,* A*=Atlantic-group,* S1*= Spain1-group,* S2*= Spain2-group, A-F-cluster, F-A-cluster*.

| BAPS cluster |  | F | A | S1 | S2 | A-F |
| --- | --- | --- | --- | --- | --- | --- |
| A |  | 0.672*** | - |  |  |  |
| S1 |  | 0.561*** | 0.491*** | - |  |  |
| S2 |  | 0.484*** | 0.510*** | 0.216*** | - |  |
| A-F |  | 0.619*** | 0.645*** | 0.265*** | 0.224*** | - |
| F-A |  | 0.704*** | 0.722*** | 0.315*** | 0.303*** | 0.296*** |

Significance obtained by 10,100 permutations: *** = P<0.001.

Supporting information Appendix S6.

Matrix of pairwise F_ST_ values among 27 studied populations in *Festuca rubra*. The pairwise F_ST_ values are below diagonal and p-values are above diagonal.

Supporting information Appendix S7.

Endophyte infection incidence (E+) and ploidy level frequencies in *Festuca rubra* in each genetic cluster at K=6 (N=566) as obtained by BAPS. Clusters: A=*Atlantic-group*, *F=Fennoscandia-group, S1=Spain1-group, S2= Spain2-group, F-A-cluster, A-F-cluster.* Ploidy levels: tetraploids: 2*n*=4x=28; hexaploids: 2*n*=6x=42; and octoploids: 2*n*=8x=56.

|  |  | E+ | Ploidy level | | | | |
| --- | --- | --- | --- | --- | --- | --- | --- |
| Cluster | N | % | 2*n*=28 % |  | 2*n*=42 % |  | 2*n*=56  % |
| K=6 |  |  |  |  |  |  |  |
| A | 181 | 35.9 | 0.6 |  | 95.6 |  | 3.9 |
| F | 218 | 40.8 | 0.5 |  | 92.2 |  | 7.3 |
| S1 | 53 | 57.0 | 50.9 |  | 49.1 |  | 0 |
| S2 | 69 | 73.9 | 55.1 |  | 37.7 |  | 7.2 |
| A-F | 23 | 43.5 | 17.4 |  | 78.3 |  | 4.3 |
| F-A | 22 | 40.9 | 4.5 |  | 95.5 |  | 0 |

Supporting information Appendix S8.

The numbers of individuals with different ploidy levels in North-Atlantic (NA), Fennoscandia (FE) and Spain (SP) among different BAPS clusters. BAPS clusters: *A=Atlantic-group*, *F=Fennoscandia-group*, *S1=Spain1-group*, S2= *Spain2-group*, *F-A-cluster*, *A-F-cluster.* Ploidy levels: tetraploids: 2*n*=4x=28; hexaploids: 2*n*=6x=42; and octoploids: 2*n*=8x=56.

|  | *A* | |  | *F* | |  | *S1* | | |  | *S2* | | |  | *A-F* | | |  | *F-A* | |
| --- | --- | --- | --- | --- | --- | --- | --- | --- | --- | --- | --- | --- | --- | --- | --- | --- | --- | --- | --- | --- |
|  | NA | FE |  | NA | FE |  | SP | NA | FE |  | SP | NA | FE |  | SP | NA | FE |  | NA | FE |
| Tetraploids | 1 | 0 |  | 1 | 1 |  | 27 | 0 | 0 |  | 38 | 0 | 0 |  | 1 | 2 | 1 |  | 1 | 0 |
| Hexaploids | 165 | 8 |  | 5 | 196 |  | 2 | 4 | 18 |  | 7 | 11 | 8 |  | 0 | 13 | 5 |  | 7 | 14 |
| Octoploids | 6 | 1 |  | 0 | 16 |  | 0 | 0 | 0 |  | 5 | 0 | 0 |  | 0 | 1 | 0 |  | 0 | 0 |

Supporting Information Appendix S9.

All data of individual samples including BAPS cluster assignments, endophyte statuses, ploidy levels and allele
